# An Efficient Hardware-Oriented Single-Pass Approach for Connected Component Analysis

**DOI:** 10.3390/s19143055

**Published:** 2019-07-11

**Authors:** Fanny Spagnolo, Stefania Perri, Pasquale Corsonello

**Affiliations:** 1 Department of Informatics, Modeling, Electronics and System Engineering, University of Calabria, 87036 Rende, Italy; 2 Department of Mechanical, Energy and Management Engineering, University of Calabria, 87036 Rende, Italy

**Keywords:** connected component analysis, features extraction, FPGAs, embedded systems

## Abstract

Connected Component Analysis (CCA) plays an important role in several image analysis and pattern recognition algorithms. Being one of the most time-consuming tasks in such applications, specific hardware accelerator for the CCA are highly desirable. As its main characteristic, the design of such an accelerator must be able to complete a run-time process of the input image frame without suspending the input streaming data-flow, by using a reasonable amount of hardware resources. This paper presents a new approach that allows virtually any feature of interest to be extracted in a single-pass from the input image frames. The proposed method has been validated by a proper system hardware implemented in a complete heterogeneous design, within a Xilinx Zynq-7000 Field Programmable Gate Array (FPGA) System on Chip (SoC) device. For processing 640 × 480 input image resolution, only 760 LUTs and 787 FFs were required. Moreover, a frame-rate of ~325 fps and a throughput of 95.37 Mp/s were achieved. When compared to several recent competitors, the proposed design exhibits the most favorable performance-resources trade-off.

## 1. Introduction

In recent years, advances in embedded vision systems led to the implementation of compact smart cameras. Smart cameras are machine vision systems that, in addition to sensors that capture images, provide the capability of extracting application-specific information from captured images. These embedded systems equipped with camera sensors represent efficient on-board solutions for several commercial applications, ranging from Advanced Driver-Assistance Systems (ADAS) to automated surveillance systems [1,2]. Computer vision tasks that lie behind these applications are very computationally intensive, often requiring special-purpose solutions to ensure real-time performances and low-power dissipation. The Connected Component Analysis (CCA) is one of the above-mentioned tasks, and it is very frequently used in several image analysis and pattern recognition algorithms [3]. The main goal of CCA is to extract connected components in a binary image, and synthetic data, such as area, bounding boxes, center of gravity etc., which will be then further processed depending on the specific application. In traditional software implementations, CCA is the combination of two subsequent computations: Connected Component Labeling (CCL) and Features Computation (FC). The CCL process allows different connected components to be distinguished in the input binary image by assigning a unique label to all pixels that belong to the same connected component [4]. Then, in order to extract one or more of the above-mentioned synthetic data parameters required by the subsequent processes, the FC algorithm elaborates such labeled images. Mainly, the CCL process scans the input image in a raster order to label each foreground pixel depending on the labels already assigned to the pixels within a chosen neighborhood typically setting either 4-connectivity or 8- connectivity [5]. In the presence of complex geometric shapes of connected components, CCL can require sequential operations to properly manage critical events, which are also called label collisions. The latter occur when differently labeled pixels are actually part of the same connected component. In order to resolve collisions, flagging the colliding labels as equivalent to each other, typical CCL algorithms require multiple sequential raster scans of the input binary image, which makes the CCL, and, consequently, the CCA, which is a very time-consuming task [6].

However, in many applications, only the specific features produced by the FC computation are actually subsequently processed [1,7,8,9,10,11,12,13], which makes the intermediate output of the CCL step not strictly necessary. As an example, extracting the area of the connected components is fundamental in the medical field for classification of blood infections [7] and cancer detection [8,9], as well as for automotive [1] and space [12] applications. In these contexts, multiple image scans required to fully complete the CCL step can be avoided [14] and one-scan CCA algorithms can be exploited to achieve higher performances [15]. Such a strategy is especially convenient when custom hardware architectures are adopted.

Hardware architectures for CCA computation are highly demanded in all application fields where high-speed, resource efficiency, and low-power consumption are mandatory constraints. Most often, they are adopted in modern design solutions where embedded systems equipped with high-resolution camera sensors are used to realize fast memory-efficient streamed-oriented systems [1,2].

This paper presents a novel single-pass CCA algorithm and a low-cost high-performance custom hardware architecture suitable for its implementation. The custom circuit proposed in this case has been purposely designed with embedded capability for the development of a smart camera device to be accommodated within modern heterogeneous FPGA-based systems. As is well known, such realization platforms represent an attractive solution to achieve synergy between hardware and software computing resources. When implemented in a Xilinx XC7Z020 FPGA chip, the proposed architecture allows a frame rate of 325.5 fps to be reached for 640 × 480 image resolutions, running at 100MHz, and occupying only 760 LUTs and 787 FFs. When compared to several recently demonstrated architectures, the proposed implementation allows the theoretically highest possible throughput of one pixel per clock cycle to be achieved, in conjunction with a storage resources efficiency ≈ 41.2% higher than the best competitor.

## 2. Related Works and Background

**Figure 1:**
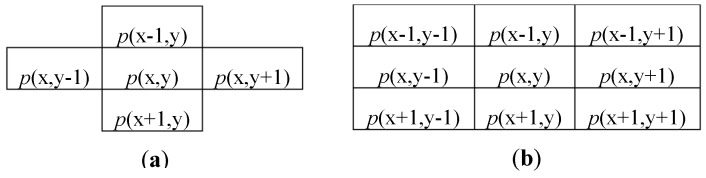
Pixel connectivity for a four-connected (**a**) and an eight-connected (**b**) set.

**Figure 2:**
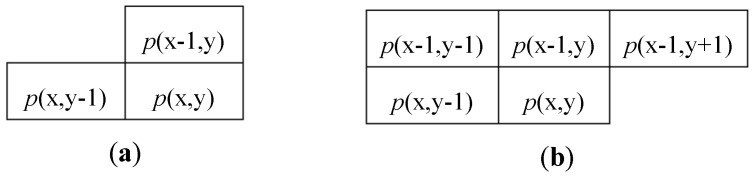
A 4-connected (**a**) and an 8-connected (**b**) neighborhood for raster scan-based CCL.

As mentioned above, the CCA can be seen as the combination of two subsequent computations: a labeling action (CCL) and a features extraction (FC). In order to assign a unique label to all pixels that belong to the same object, the CCL scans the input image in raster order and processes each pixel *p*(x,y) with its neighborhood. Typical neighbors consist of 4-connected or 8-connected pixels [5], as shown in Figure 1. Considering that the input image is streamed in raster order, it is easy to understand that, when the pixel *p*(x,y) is processed, only the pixels located within previous rows and columns are actually available. Therefore, the connectivity changes are depicted in Figure 2. Then, a new label is assigned to each newly encountered unconnected foreground pixel *p*(x,y). In the presence of only one neighbor labeled pixel, such a label is connected also to *p*(x,y), whereas, if two neighbor pixels are labeled differently, a collision occurs.

**Figure 3:**
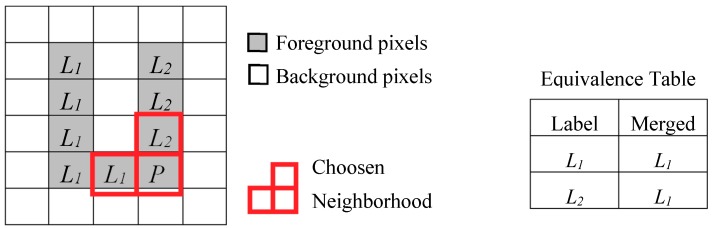
Labeling process for a U-shaped connected component.

Referring to the image depicted in Figure 3, during the scan of the first four rows, no information is provided about the connectivity between the two bars of the U-shaped connected component until the labeling of the pixel *P* takes place. Due to this, the foreground pixels scanned before *P* are associated with different labels, *L*_1_ and *L*_2_. Consequently, the neighborhood of *P*, when reached, will contain two pixels labeled as *L*_1_ and *L*_2_, which causes a collision. When the above event occurs, the label equivalence can be stored in the so-called *Equivalence Table*, which is also illustrated in Figure 3. In this case, the original label is associated with the merged label to be used in the subsequent steps.

**Figure 4:**
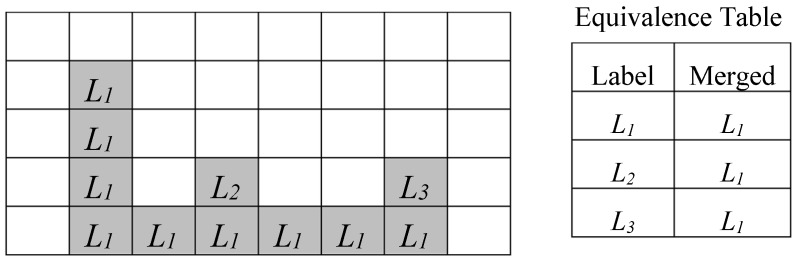
Labeling process for a more complex connected component.

In Figure 4, a more complex case is illustrated where multiple collisions have been detected. Due to such cases, the time required by the labeling process is pattern-dependent and it increases with the complexity of the connected components identified within the input binary image, since it can require multiple scans of the input image [4]. Several approaches known in literature can reduce the overall computational time by limiting the image scans required during the CCL to two [16,17,18,19,20,21]. During the first scan, a provisional labeling is performed, while equivalences among colliding labels are stored to solve collisions afterwards. The execution of the second image scan allows us to assign to all equivalent labels their final values. The efficient CCL architectures presented in References [22,23] allow the time needed to solve label collisions to be completely or partially hidden by image transfer activities that are always required in real-world heterogeneous FPGA systems. Such a strategy avoids the second image scan and improves the overall performance of the system.

**Figure 5:**
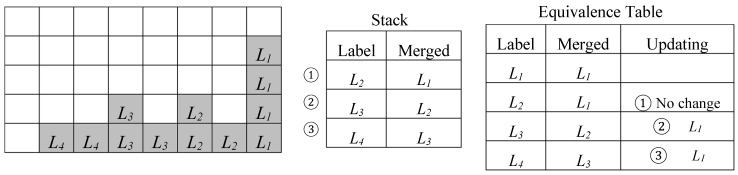
Example of the chain of collisions solved by the backward merging technique.

When the intermediate output of the CCL is not further processed, except for the FC, one-scan algorithms are the best candidates [14,24,25,26]. The example of Figure 5 illustrates how the problem of resolving the chain of collisions *L*_4_ → *L*_3_ → *L*_2_ → *L*_1_ is addressed in Reference [14]. The so-called backward merging technique, stores collisions encountered in each row of the binary image into a stack in the reverse order with which they occur. At the end of each scan line, these collisions are read from the stack and processed to modify the Equivalence Table by substituting equivalent labels with their representative smallest one. It is worth noting that this technique requires three clock cycles per equivalence, which often represents a bottleneck in streamed image processing. The approaches proposed in References [24,25] reduce memory resource requirements by either recycling after every row or efficiently processing stale labels. However, both strategies introduce a time overhead up to 20%.

Higher throughput rates are achieved with the techniques presented in References [26,27,28,29,30]. In References [27,28], run-length encoding techniques are exploited for labeling connected components while representing equivalences with linked lists. Therefore, the resolution of equivalences involves only pointer redirection operations and removes the chaining problem. The pipeline architecture proposed in Reference [29] removes the blanking period for label merging by dividing the operations into two stages, but it requires twice the memory resources.

Conversely, the strategy proposed in Reference [30] exploits an optimized row buffer, designed in the form of a shift-register, which allows the label merging to be performed on the fly, without introducing additional time. Parallel hardware implementations of one-scan CCA algorithms have been proposed in References [31,32]. The input binary image is divided into vertical slices that are parallel processed. Despite the high performances achieved, these solutions require much more hardware resources.

## 3. The Proposed Algorithm

The single-pass solution proposed, in this case, is based on runtime processing input binary pixels, by simultaneously assigning provisional labels, managing equivalent labels, and updating features data. The whole process makes use of auxiliary tables to take into account equivalences that occurred during the scan to store temporary features information. Such tables are named Translator LUT (TL) and Features Table (FT), and their size depends on the maximum number of labels the architecture is allowed to assign, which is, in this case, indicated with N_L_. Without a loss of generality, in the rest of the paper, we will focus on the extraction of the area of connected components detected in the input image, but the same approach can be used for other features, such as the bounding box and the center of gravity. Clearly, in these cases, the management of the auxiliary tables changes accordingly.

**Figure 6:**
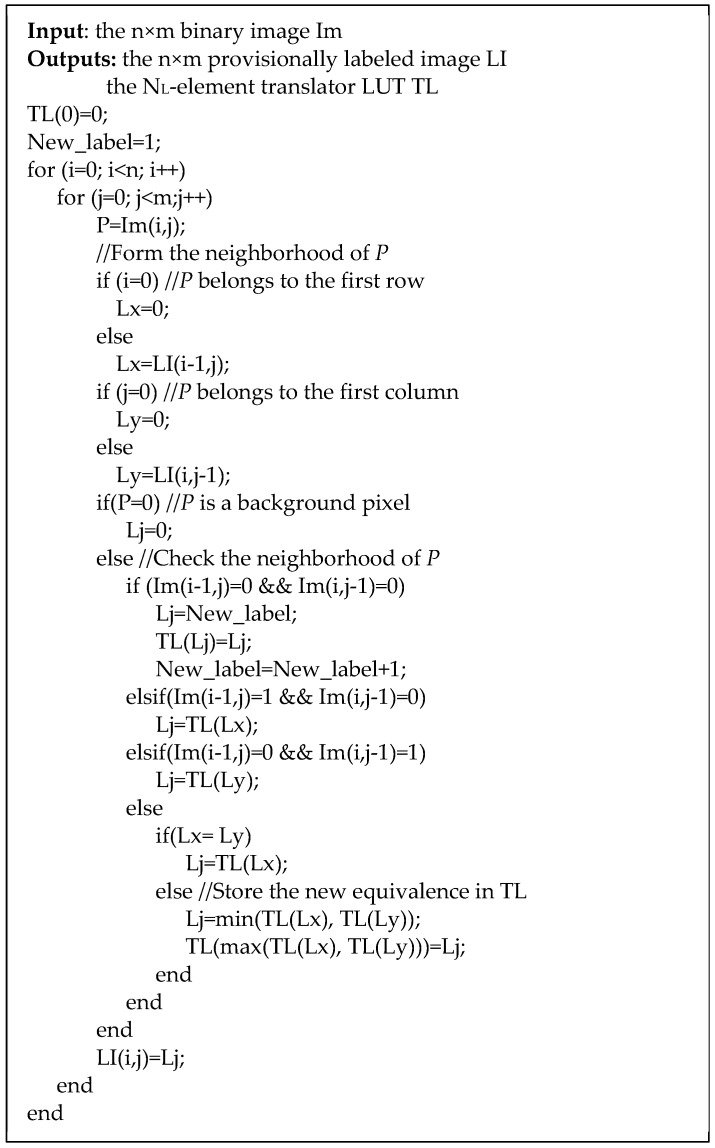
Pseudo-code of the proposed labeling technique.

The pseudo-code reported in Figure 6 describes the proposed labeling algorithm. It can be seen that each background pixel is zero-labeled, whereas, when a foreground pixel P is inputted, its 4-connected neighborhood is evaluated. Then, if P is surrounded by background pixels, it receives a new generated label Lj (with j = 1, …, N_L_ − 1). The Translator LUT (TL) is updated so that TL(Lj) = Lj. If the current neighborhood is instead composed by just one foreground pixel already labeled. Such a label is used as an address to access the Translator LUT, and the translated label is assigned to P. In the critical situations, the neighborhood contains two foreground pixels, associated to two colliding labels Lx and Ly. In such a case, the minimum between TL(Lx) and TL(Ly) is assigned to P. This ensures that: (i) the correct translated label is always propagated in the labeled image, (ii) no long chains are generated within the Translator LUT since, in cases like the one illustrated in Figure 4, propagating the minimum assigns the correct label to P. Furthermore, to update the Translator LUT with the newly discovered equivalence, TL(max[TL(Lx), TL(Ly)]) is set to min[TL(Lx), TL(Ly)]. In this way, multiple complex chains of colliding labels are avoided.

The approach adopted to manage the table FT is similar to what is mentioned above. When no collision is detected, and the generic label TL(Lj) has been assigned to the current pixel, the Translator LUT is again accessed to resume TL(TL(Lj)). This allows taking into account that, due to a prior collision, the label Lj may have been recognized as equivalent to another label, which causes an update of the Translator LUT. Then, to increase the area pixel count of the detected object, FT(TL(TL(Lj))) is incremented by one. Conversely, when a collision between TL(Lx) and TL(Ly) is detected, the minimum (Lmin) and the maximum (Lmax) among TL(TL(Lx)) and TL(TL(Ly)) are calculated. This allows us to identify those cases in which: (i) Lx is greater (smaller) than Ly, while TL(Lx) is smaller (greater) than TL(Ly), and (ii) colliding labels Lx and Ly have been recognized in previous collisions as equivalent to the same label. If Lmin = Lmax, the content FT(Lmin) is simply incremented by one, otherwise FT(Lmin) + FT(Lmax) + 1 is stored in FT(Lmin) and FT(Lmax) is zeroed. The process continues until all the input binary pixels are processed.

**Figure 7:**
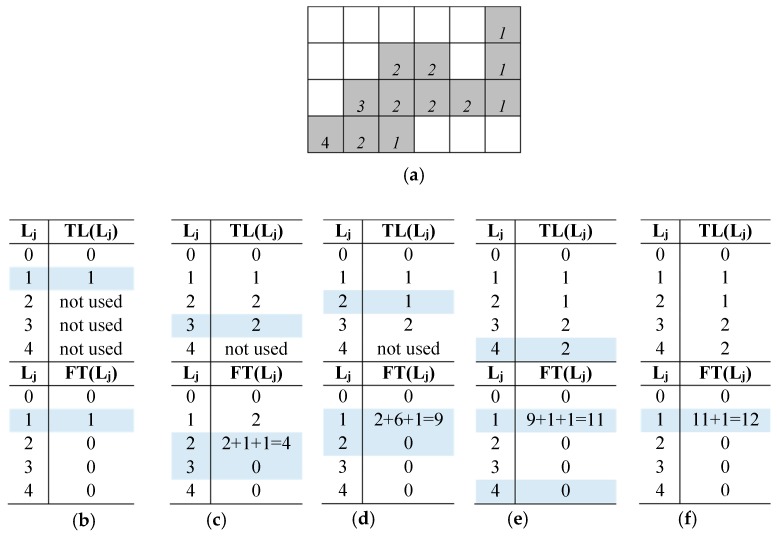
(**a**) The input binary image labeled by the novel algorithm. Status evolution of the *Translator LUT* and *Feature Table* (**b**–**f**).

To better clarify the running of the proposed algorithm, let us examine the example reported in Figure 7a. After the scan of the first row of the input image is completed, TL(1) = 1, while FT(1) = 1 because only one foreground pixel with label 1 has been counted (Figure 7b). The scan of the second row does not introduce particular conditions to be managed. Then, for the second foreground pixel in the third row, a collision between Ly = 2 and Lx = 3 occurs. The minimum label between TL(Ly) = 2 and TL(Lx) = 3 is now assigned to the current pixel. Then, as shown in Figure 7c, the Translator LUT and the Features Table are updated by writing TL(3) = 2, FT(TL(TL(2))) = FT(TL(TL(3))) + FT(TL(TL(2))) + 1 = 4. Lastly, the pixel count previously assigned to label 3 (i.e., FT(TL(TL(3)))) is zeroed. Figure 7d details the tables update when the next collision (1,2) occurs. It is important to note that, because of the previous collision between labels 2 and 3, the count referred to the latter is now transferred to the connected component identified by label 1. Figure 7e–f illustrate the last two significant occurrences in the process. It can be seen that the last foreground pixel is surrounded by pixels labeled with 2. In this case, the adopted strategy assigns TL(2) = 1 to the current pixel, which interrupts the propagation of incorrect labels and the formation of unmanageable collision chains.

**Figure 8:**
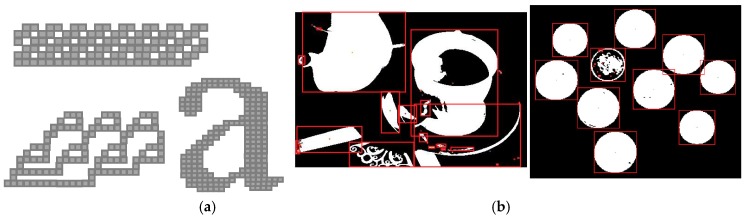
Sample images used to extract: (**a**) area features, (**b**) bounding boxes, and centroids.

In order to verify the correctness of the proposed approach, several tests were performed on software routines purposely written to model our algorithm in the MATLAB tool environment. Hundreds of benchmarks with different sizes and patterns have been processed to extract the area feature, the bounding boxes, the centroid, and more, for each recognized connected component. Figure 8a illustrates some of synthetic critical test patterns. In such cases, complex collision chains occur. Figure 8b shows sample images from which bounding boxes and centroids have been extracted. In all the examined cases, the features extracted by exploiting the proposed algorithm perfectly match with those produced by using the functions (e.g., *bwlabel* and *regionprops*) available in the MATLAB Image Processing Toolbox. This demonstrates that, as expected, the algorithmic-level optimizations introduced do not affect the quality of the CCA decisions with respect to the conventional approach.

## 4. The Proposed Architecture

**Figure 9:**
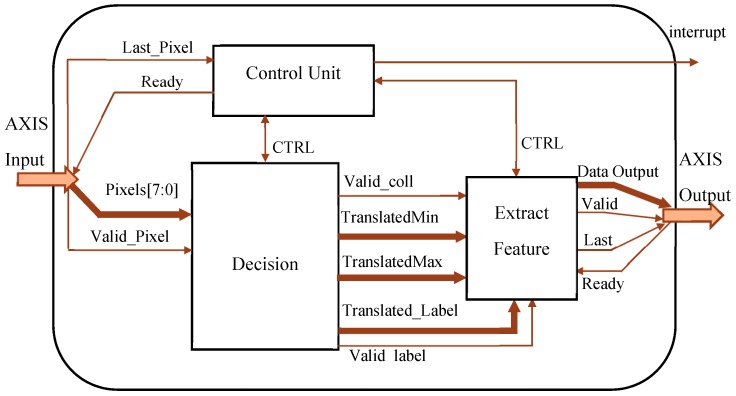
Top-level architecture of the proposed CCA accelerator.

Figure 9 illustrates the top-level architecture of the realized circuit. It has been designed to be included in a heterogeneous FPGA-based system. Therefore, input and output interfaces were realized to sustain standard AXI transactions [33]. Furthermore, an interruption on the completion signal for the host processor is generated. All internal data buses are endowed with appropriate auxiliary signals used to assert data validity. The external data dispatcher establishes if the CCA circuit is ready to process input pixels through the *Ready* signal received from the *Control Unit*, which orchestrates the whole running of the architecture until the last pixel of the input frame is received. The latter condition is identified through the appropriate *Last_pixel* and *Valid_Pixel* signaling.

Details about the implementation and running of the modules mentioned above are provided in the following sub-sections.

The *Decision* module, which accommodates the *Translator LUT*, transfers information concerning assigned and equivalent labels to the *Extract Feature* module through the *Translated_Label*, *TranslatedMin*, and *TranslatedMax* data buses. The latter are *nbl*-bit wide, with $nbl=\left\lceil log_{2}\left(N_{L}-1\right)\right\rceil$. When the last pixel of the input image has been processed in raster order and features of interest are available, an interrupt on completion is generated. Then, features are sent for subsequent elaborations, while the whole architecture is reset. For our specific application, results are transferred through an output AXI-Stream interface.

### 4.1. The Decision Module

**Figure 10:**
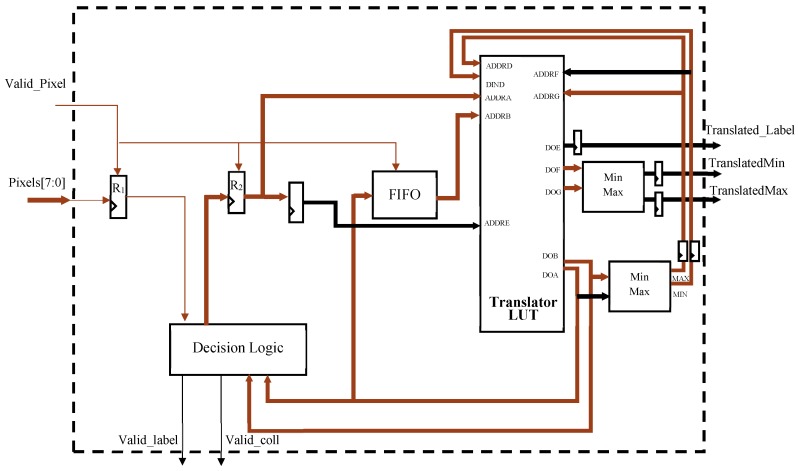
Design of the Decision module.

As illustrated in Figure 10, the decision module is composed of a line buffer, the Translator LUT, and a decision logic. The former, which consists of two registers (R_1_, R_2_) and a FIFO, locally stores the provisional labels already assigned to previously scanned pixels and required for correctly arranging a four-connected neighborhood at each clock cycle. It is worth noting that the FIFO stores at most m-1 labeled pixels, with m being the number of columns in the input image. Since background and foreground incoming pixels are assumed being equal to 0 and 255, respectively, R_1_ is a simple flip-flop storing the LSB of the 8-bit input pixel. In order to establish if a label must be assigned or not to the current pixel, the decision logic evaluate the content of R1 and the labels, if any, assigned to the neighboring pixels and are available within the register R_2_ and at output of the FIFO. As visible in Figure 8, these labels are directly connected to the inputs ADDRA and ADDRB of the multi-port memory block acting as Translator LUT. Corresponding asynchronous outputs DOA and DOB are sent to the decision logic block that implements the rules detailed in the previous section. Such a block assigns the correct label to the current pixel, which is then stored in R_2_. In the next clock cycle, the translated value of the label stored in R_2_ enters the FIFO. This strategy avoids label collision chains to be left unresolved. When a collision is detected, the Translator LUT needs to be updated, as discussed in Section 3. To this purpose, the minimum (MIN) and maximum (MAX) between DOA and DOB are calculated and, in the next clock cycle, the Translator LUT is updated through the DIND port, by writing TL(MAX) = MIN. This information is then transferred to the Extract Features block after a second access to the Translator LUT, performed in a pipeline fashion, through the ports ADDRF and ADDRG. From Figure 8, it can be seen that, after a minimum-maximum calculation, DOF and DOG, are outputted as TranslatedMin and TranslatedMax. Furthermore, the translated content of R_2_, made available through the output port DOE, is opportunely delayed and then sent to the Extract Features block by the data bus named Translated Label.

### 4.2. The Feature Extract Module

**Figure 11:**
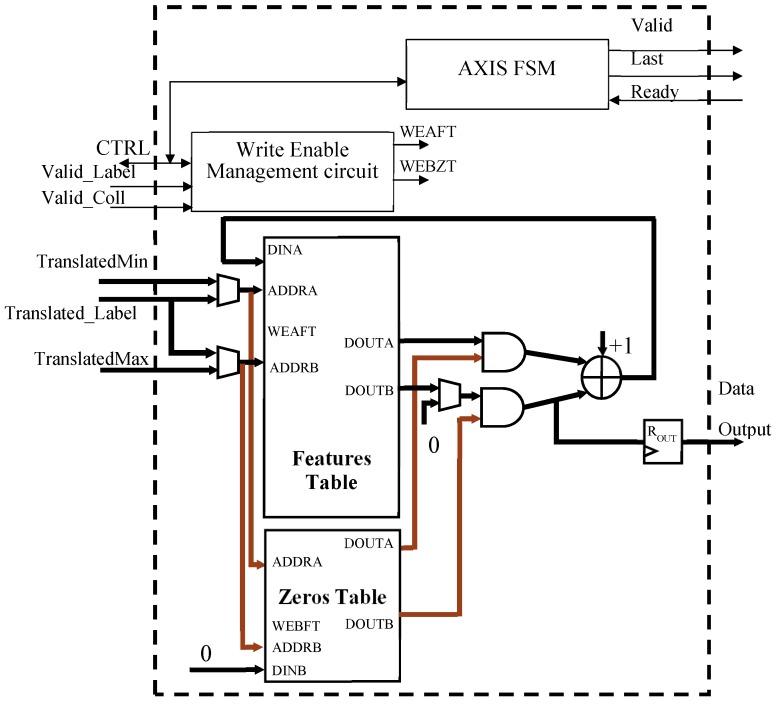
Design of the Extract Feature module.

The conceptual design of the Extract Feature module is depicted in Figure 11. It consists of the Features Table and a Zeros Table, besides the control circuits used for generating write enable (WEAFT and WEBZT) signals and AXIS protocol signals. The Zeros Table is implemented as a one-bit Simple Dual Port memory of size N_L_, and it is initialized to 1. Such a memory intervenes in the case of a collision between TranslatedMin and TranslatedMax. As described in Section 3, in this case, the position FT(TranslatedMax) must be reset, while the updated count is written in FT(TranslatedMin). To perform this action, its TranslatedMax location is zeroed through the port DINB.

When a valid translated label appears on the Translated_Label bus, the area consistency of the connected component associated with that label is read by means of the port ADDRA of the Features Table. It is incremented by one and rewritten through the same port during the next clock cycle. On the other side, when a valid collision between TranslatedMin and TranslatedMax is detected, the Features Table is asynchronously accessed through the ports ADDRA and ADDRB. Its contents outputted on DOUTA and DOUTB are AND-ed with the Zeros Table corresponding data and summed, incremented by 1 and, lastly, written in the next clock cycle through the port DINA.

When all pixels are treated, the Control Unit activates the AXIS FSM that scans the Features Table, transmits results over the AXIS-Stream interface, and simultaneously re-initializes the memory for the subsequent elaboration. It is worth noting that, while in the referred implementation, we used an AXI-Stream output interface. For several simpler applications, a cheaper AXI-Lite interface could suffice, which further saves logic resources.

## 5. Experimental Results

**Figure 12:**
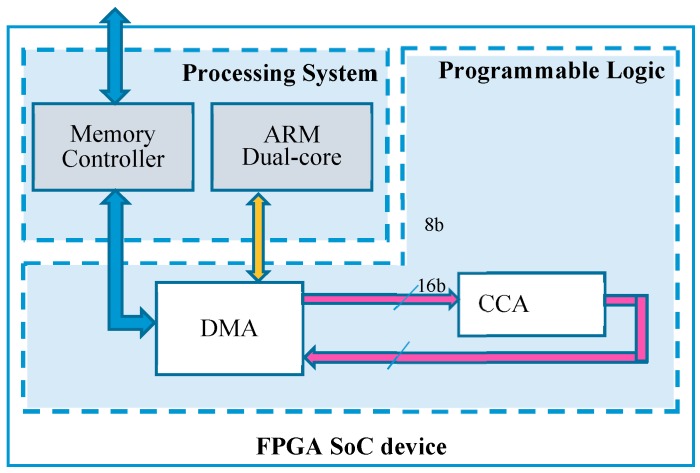
Integrating the proposed CCA accelerator within modern heterogeneous FPGA-based SoCs.

The architecture described above has been realized on a heterogeneous FPGA-based SoC Zynq-7000 [34]. Figure 12 illustrates the schematic diagram of the heterogeneous embedded system realized as test setup. The Xilinx Direct Memory Access (DMA) module [35] is used to transfer data from the external memory to the CCA core and vice versa. The DMA exploits its Advance eXtensible Interface (AXI) memory-mapped interfaces to communicate with the external memory through the memory controller of the Processing System (PS). Conversely, data transfers to/from the custom core follow the AXI-Stream standard protocol. Table 1 summarizes results obtained by the proposed CCA architecture and by other implementations existing in the literature. It is worth pointing out that none of the cited works implements AXI interfaces for direct inclusion in heterogeneous FPGA-based systems. It is also important to note that, to improve resources efficiency, all internal memory banks are implemented by using 352 LUTs of distributed RAM, instead of Block-RAM. If Bounding Boxes are the features of interest, 176 more LUTs of distributed RAM for the *Features Table* are required to accommodate the coordinates of connected components instead of their area pixel counts.

Since different FPGA device families were used in References [24,25,27], which is a direct comparison with our CCA accelerator in terms of speed performances and resources requirements that would be unreliable. However, the capability, i.e., the number of managed labels *N_L_*, and the number of clock cycles per pixel, also reported in Table 1, are not related to the prototyping platform used. For what concerns the number of clock cycles per pixel, it can be observed that the proposed design, as well as the architecture presented in Reference [27], reaches a unitary throughput, which is significantly higher than References [24,25], independently of the used technology.

For a fair discussion, we introduce the resource efficiency parameter as defined in Equation (1). In this case, the maximum number of labels *N_L_* is related to the amount of resources, expressed in terms of kbits, and multiplied by the number of clock cycles per pixel.

(1) $$Resource_{EFF}=\frac{N_{L}\times 1024}{Resource\;\left[kb\right]\times \frac{Cycles}{Pixel}}$$

**Figure 13:**
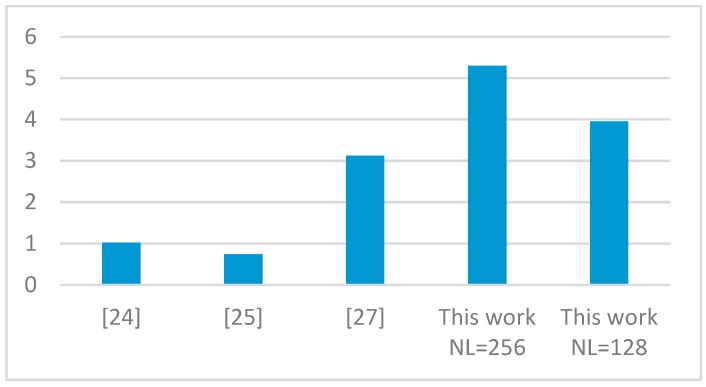
Comparison of the resource efficiencies.

In this way, all terms referenced in Equation (1) are made independent of the technology. In fact, the resources occupancy, measured in terms of kbits, is calculated by differently weighting the FPGA resources to take into account the specific technology used. While the Artix-7 chip used in this work and the Kintex-7 device used in Reference [25], being part of the Xilinx Series-7 family, make six-input LUTs available, the Virtex-2 device used in References [24,27] provide four-input LUTs. Thus, this differently affects the kbits related to the total amount of resources occupancy. Conversely, flip-flops and on-chip memory contribute to the total amount of kbits independently of the technology used. Figure 13 plots the resource efficiency achieved by the compared designs. Obtained results demonstrate that the proposed implementation, with its higher efficiency, allows the best trade-off between hardware requirements, speed performances, and computational capability to be achieved.

Memory requirements are further analyzed in Table 2, where the number of bits used to represent labels and features are indicated with *nbl* and *nbf*, respectively. Such a comparison shows that, in contrast to References [24,25], the novel architecture does not need the merger table since the *Translator LUT* is made able to implement both merging and translation operations. It is also worth noting that, unlike the CCA architectures demonstrated in References [25,27], the algorithm proposed, in this case, does not need further auxiliary tables.

**Figure 14:**
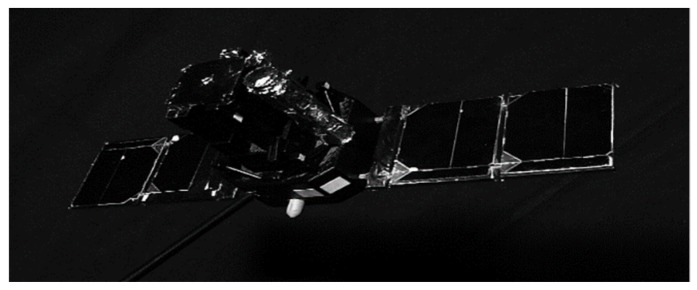
Sample test image.

The proposed hardware accelerator has been integrated in a complete image processing system for aerospace applications. High-resolution input images are first filtered, thresholded, and binarized. Then Regions-of-Interest (ROIs) are transferred to the CCA circuit for being processed at a rate higher than 250 fps. An example of the input frame is depicted in Figure 14. A dataset of 204 benchmark frames have been thoroughly analyzed to verify the appropriateness of all design parameters.

**Figure 15:**
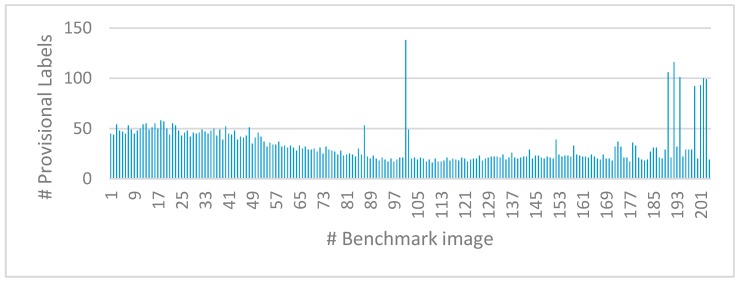
Number of provisional labels assigned by the proposed algorithm to the examined image dataset.

As an example, Figure 15 shows the number of labels assigned by the proposed CCA approach to the processed benchmark images, numbered from 1 to 204 along the *x*-axis. It can be easily seen that *N_L_* = 256 satisfies the specific application requirements. Furthermore, from the same experiments, we found that the largest possible connected component spans over several thousand pixels, which makes the 16-bit data width more than appropriate for the *Features Table* memory.

Just for a comparison purpose, we measured the speed performance achievable by an all-software implementation of the novel CCA approach. Even though it benefits from several algorithmic improvements with respect to traditional approaches, when executed by the dual-core ARM A9 processor at the 666 MHz frequency, it reaches a maximum frame rate of ~200 fps. Conversely, the CCA accelerator presented in this work exhibits a frame rate that outperforms its all-software counterpart by ~62%.

## 6. Conclusions

A novel CCA method and its hardware design have been presented. The custom circuit has been implemented within a Xilinx Zynq XC7Z020 FPGA SoC toward the realization of a complete heterogeneous embedded system. Experimental tests have demonstrated that the proposed solution allows the highest possible throughput of one cycle per pixel to be reached. When processing 640 × 480 images at the 100 MHz running frequency, the novel circuit achieves a frame rate of 325.5 fps. Thanks to the optimization strategies adopted in this case, when compared to several competitive CCA hardware implementations known in literature, the proposed CCA accelerator also exhibits the highest resource efficiency.

## Figures and Tables

**Table 1:** Characteristics of the proposed CCA implementation and other state-of-the-art designs.

	Ma et al. [24]	Klaiber et al. [25]	Tang et al. [27]	This Work	
Technology	Virtex II (150 nm)	Kintex-7 (28 nm)	Virtex II (150 nm)	XC7Z020 (28 nm)	
Image Size	640 × 480	256 × 256	640 × 480	640 × 480	
Feature	A ^1^	BB ^2^	BB	A	A
N_L_	128	130	320	256	128
LUTs	1757	493	654	760	506
FFs	600	296	227	787	774
BRAM [kb]	72	108	92	0	0
*f* [MHz]	40.64	185.59	97.07	100	140
Cycles/Pixel	1.25	1.25	1	1	1
Mp/s	31	141.59	92.57	95.37	133.5

^1^ Area, ^2^ Bounding boxes.

**Table 2:** Memory requirement of several CCA architectures processing n × m input images.

Resource	Memory Requirements (bits)			
	Ma et al. [24]	Klaiber et al. [25]	Tang et al. [27]	This Work
Row Buffer *RB*	m×nbl	$\left\lceil \frac{m}{2}\right\rceil \times nbl$	2×m	1+(m−2)×nbl
Collision Table *S*	$N_{L}\times nbl$	m×nbl	-	-
Merger Table *MT*	m×nbl	$N_{L}\times (nbl+\;\left\lceil log_{2}\left(n\right)\right\rceil$)	-	-
Translator LUT *TL*	$N_{L}\times nbl$	-	-	$N_{L}\times nbl$
Reuse FIFO *R*	-	$N_{L}\times nbl$	-	-
Label Stack *LS*	-	$m\times \left\lceil \frac{nbl}{10}\right\rceil$	-	-
Valid flags *V*	-	nbl	-	-
Active tags *E*	-	m	-	-
Next Table *NT*	-	-	$N_{L}\times nbl$	-
Head Table *HT*	-	-	$N_{L}\times nbl$	-
Tail Table *TLT*	-	-	$N_{L}\times nbl$	-
Data Table *DT*	$2\times N_{L}\times nbf$	$N_{L}\times nbf$	$N_{L}\times nbf$	$N_{L}\times nbf$
